# Mothers’ perceptions and experiences of caring for sick newborns in Newborn Care Units in public hospitals in Eastern Uganda: a qualitative study

**DOI:** 10.1186/s12978-023-01649-1

**Published:** 2023-07-20

**Authors:** Phillip Wanduru, Claudia Hanson, Peter Waiswa, Angelina Kakooza-Mwesige, Helle Molsted Alvesson

**Affiliations:** 1 Department of Global Public Health, Karolinska Institutet, Stockholm, Sweden; 2 Department of Health Policy, Planning, and Management, School of Public Health, Makerere University, Kampala, Uganda; 3 Department of Disease Control, London School of Hygiene and Tropical Medicine, London, England; 4 Department of Paediatrics and Child Health, School of Medicine, Makerere University, Kampala, Uganda

**Keywords:** Mothers’ participation, Newborn care, Newborn Care Units, Uganda, Qualitative interviews, Observations

## Abstract

**Introduction:**

Mothers’ participation in the care of their sick newborns in Newborn Care Units (NCUs) has been linked to several advantages including earlier discharge, fewer complications, better mother–baby bonding, and an easier transition to home after discharge. This study aimed to understand mothers’ perceptions and experiences while participating in the care of their sick newborns in the NCUs to inform interventions promoting mothers’ participation in public health facilities in Uganda.

**Methods:**

We conducted an exploratory qualitative study comprised of 18 in-depth interviews with mothers caring for their newborns in two NCUs at a Regional Referral and General hospital in Eastern Uganda between April and May 2022. The interviews were audio-recorded and then transcribed. For analysis, we used a thematic analysis approach.

**Results:**

The fear of losing their baby was an overarching theme that underlay mothers’ perceptions, actions, and experiences in the NCU. Mothers’ confidence in the care provided to their babies was based on their baby’s outcomes. For example, when mothers saw almost immediate improvement after treatment, they felt more confident in the care than when this was not the case. Furthermore, mothers considered it essential that health care providers responded quickly in an emergency. Moreover, they expressed concerns about a lack of control over their personal space in the crowded NCU. Additionally, caring for babies in these settings is physically and financially taxing, with mothers requiring the combined efforts of family members to help them cope.

**Conclusion:**

This study shows that for mothers of sick newborns in the NCU, the baby’s survival is the first concern and the basis of mothers’ confidence in the quality of care provided. Efforts to improve parental participation in NCUs must focus on lowering the costs incurred by families in caring for a baby in the NCU, addressing privacy and space concerns, leveraging the family’s role, and avoiding compromising the quality of care in the process of participation.

## Background

Newborn mortality in Sub-Saharan Africa (SSA) is estimated to be 27 per 1000 live births (2021), making it the region with the highest global burden [1]. In Uganda, newborn mortality is estimated at 18 per 1000 live births (2021), an improvement from 22 per 1000 births at the start of the Sustainable Development Goals (SDG) era in 2015, but progress is too slow to meet the SDG target of 12 per 1000 live births [1]. More than two-thirds of newborn deaths in Uganda are the result of complications occurring during birth, such as birth asphyxia and preterm birth complications [2]. In light of this situation, prioritizing the improvement of the quality of care for newborns, particularly those with complications in the first days of life, is likely to have a significant impact on the newborn mortality rate [3].

Fortunately, efforts to improve the quality of care for newborns around the time of birth have increased in recent years in Uganda [3, 4]. The World Health Organization (WHO) has also developed key guiding *s*tandards for caring for sick newborns in health facilities, which have been adopted by Uganda [5, 6]. These standards emphasize the importance of involving parents or other caregivers in the care of their sick newborns. Furthermore, numerous studies have indicated that parental participation in the care of admitted newborns is associated with better clinical outcomes, including better weight gain, early discharge, and fewer complications [7–9]. Improved bonding with the baby, an easier transition from hospital to community, and less anxiety among caregivers have also been reported [7, 9, 10].

In many Low and Middle-Income Countries like Uganda, investments in health are typically focused on ensuring the availability of drugs and equipment, addressing human resource capacity deficits, and improving data systems [11]. Little consideration is given to social or people-centred aspects, such as how to support and integrate parents in the care of their hospitalized newborns [4, 11]. For example, the Ugandan national guidelines do not cover parents’ or caregivers’ involvement in the Newborn Care Units (NCUs) where sick babies will be admitted after birth [12, 13]. Despite the lack of official guidance, health care providers (HCPs) in NCUs ask mothers to participate in the care of their babies by carrying out tasks such as cord care, danger sign recognition, or nasal gastric tube feeding [14].

In Uganda, the Ministry of Health and partners are becoming increasingly interested in exploring ways to operationalize the integration of parents into the care of their sick babies after birth. This interest is partly motivated by the Respectful Maternity Care movement. This movement advocates for not separating sick newborns from their mothers for a variety of reasons, including the need to encourage on-demand breastfeeding and employ practices like kangaroo mother care [15–17].

With a view to informing national guideline development about how to institutionalize the participation of parents in the care of their sick babies at facilities, we reviewed the literature on caregiver experiences of participating in the care of newborns admitted to NCUs. We found that the vast majority of the research regarding parental participation was from high-income countries, with only a few studies from middle-income settings where family-centred models are in practice [18]. Notably, there is little evidence to support parental participation in the care of sick babies in lower-income settings despite these being known to have a larger burden of sick newborns. Given this gap in knowledge, in this study, we sought to understand mothers’ experiences of taking care of their babies in resource constrained NCUs in Eastern Uganda in order to inform guidance and policies supporting mothers’ involvement in similar settings.

## Materials and methods

### Study setting

This study was carried out in the NCUs of two high-volume hospitals in East Central Uganda: A General Hospital and a Regional Referral Hospital. Both General and Regional Referral hospitals offer outpatient, inpatient, and emergency care in the fields of medicine, surgery, obstetrics, and paediatrics. A Regional Referral hospital provides more specialised services than a General hospital as it employs consultant and/or senior consultant paediatricians, surgeons, doctors, and obstetricians. In terms of referral hierarchy, General hospitals refer to Regional Referral hospitals [19].

These two hospitals were chosen as research sites since they represent a typical high-volume government-run health facility in Uganda, serving mostly poor people who are unable to afford private care. Being government-run, they offer free medical care. Both hospitals manage approximately 550 births per month [19]. The NCU’s caseload amounts to 1000 and 1200 per year in the general and regional hospital respectively.

The two NCUs offer similar newborn care services including temperature support, newborn resuscitation including oxygen therapy, feeding support (including nasal gastric tube feeding), and provision of essential newborn medications like antibiotics, anti-convulsant and intravenous fluids. Stockouts of supplies, including medicines, are common in this setting [20]. Both NCUs are staffed with two to three nurses working in three 8 hourly shifts. A paediatrician and intern doctor(s) are present during the day and on-call during the night. There are no sleeping arrangements available for mothers or any other caregivers in the NCU.

### Study design

This study uses an exploratory qualitative design using in-depth interviews and observations to understand the experiences of mothers when caring for babies in the NCU.

### Study participants

Caregivers were mothers, relatives or even friends who had taken on the role of being main caregivers for the babies in the NCU for a minimum of 48 h. It was presumed that after this 48-h timeframe, the caregivers had acquired some experience to share. Notably, the selected hospitals’ NCUs exclusively had mothers serving as caregivers, leading to all participants being mothers.

Participants were selected using purposive heterogeneity sampling. We looked for variation regarding age and socioeconomic status, since existing evidence and our experience in the setting suggested that participants’ experiences would differ based on these characteristics [21]. We initially interviewed nine mothers, including younger and older mothers, as well as those in poor economic situations versus those in better economic situations. The economic situation of mothers was determined by asking nurses. Mothers who could afford food and utilities such as baby diapers without requiring hospital assistance were classified as well off, while those who could not were classified as not well off. After interviewing the first nine mothers, we conducted a preliminary analysis of the data and realized that the severity of the babies’ condition was a source of variation in mothers’ experiences. As a result, we sampled another seven mothers while taking these factors into account. So, in the end, there were sixteen mothers in total, nine from the Regional Referral Hospital and seven from the General Hospital. The demographic characteristics of mothers who participated in the study are included in Table 1.

**Table 1:** A table showing sociodemographic of mothers who participated in the study

	N = 16
Age range	
19 or less	4
20–24	4
25–30	5
30 or more	3
Marital status	
Never lived with a spouse	3
Living with spouse	10
Separated	3
Level of education	
No education	4
Primary level	4
Secondary level	5
Above secondary level	3
Number of children	
1–2	4
2–3	5
3–5	5
> 5	2
Employment status	
Professional with formal employment	4
Small business owner	4
Unemployed	8

### Data collection processes

We used an interview guide that was informed by previous research on caregivers’ experiences of caring for sick babies in Uganda [21, 22]. Aspects from research evaluating family-centred newborn care models inspired our inclusion of probes on emotional attachment during participation and the importance of the physical environment [23]. The guide included questions about (i) the mothers’ caregiving activities, (ii) their motivations, (iii) their feelings about participation, and (iv) their interactions with HCPs. The interviews were conducted by PW, a male with medical training, with the support of two female research assistants who were social scientists. All interviewers had extensive experience in collecting qualitative research data. They had also lived in the area for more than 10 years, giving them a good understanding of the participants’ culture and language. The interviews were conducted in the local language (Lusoga) and audio recorded with the participants’ consent. In addition, descriptive and reflective field notes were taken throughout data collection by research assistants. Data collection continued until data saturation was reached, which happened when participant information became repetitive on the topics of mothers’ motivation to participate, activities they participated in, their perceptions of care, and their experiences in the NCU. In addition to interviews, we conducted non-participant observations of NCU activities. Two study team members, one with medical training and the other without, undertook separate 6-h observations of the NCU operations at each hospital. The observations were guided by an observation guide developed by the research team which included aspects of general appearance of the NCU; description of the role of individuals in the NCU, highlighting those that stand out; human traffic; personal space; and other human interactions. The data collectors wrote notes on these aspects based on their observations.

### Data analysis

The audio-recorded interviews were transcribed and later translated into English by the same team that conducted the interviews, with each member transcribing and translating their own interviews. For analysis, we followed the steps of thematic analysis outlined by Braun and Clarke [24]. This process included reading through the data repeatedly to understand its meaning, coding, identifying themes, and establishing the relationships between them. Interview transcripts were coded using NVivo 11 Plus (QSR International, Memphis, USA). PW led the open coding, which included coding and presenting emerging themes at the manifest level. Following that, the research team conducted a series of discussions to review themes in order to give them meaning and identify underlying patterns [24]. These discussions also included double-checking with the codes, and transcripts were required. HMA, a medical anthropologist, guided the process.

### Ethical considerations

Ethical clearance for this study was obtained from the Makerere University School of Public Health Higher Degrees, Research and Ethics committee, (Approval number SPH-2021-126) and Uganda National Council of Sciences and Technology (Approval number: HS2057ES). Following a comprehensive explanation of the study’s objectives and methods, informed consent (signed or witnessed by a thumbprint) was obtained from all eligible participants.

## Results

Mothers’ fear of losing their baby was a pervasive sentiment that drove their participation in the NCU as well as shaped their perceptions and experiences. This overarching theme organized our analysis and supported the themes developed [25].

In the following section, we provide a detailed description of the themes and sub-themes as presented in Table 2.

**Table 2:** Summary of emerging themes and sub-themes

Overarching theme: Mothers’ fear of losing their baby was a pervasive sentiment that drove their participation in the NCU, and shaped perceptions and experiences	
Theme 1: Mothers’ perceptions of quality of care were influenced by baby outcomes and health care providers’ responsiveness	a. Mothers’ confidence in medical treatment depended on baby outcomes b. Health care providers’ responsiveness was not taken for granted
Theme 2: Mothers’ focus on “doing” things that aid their baby's survival	a. Focus on doing b. Suppressing own feelings and attachment to baby
Theme 3: Mothers’ needs in the process of caring for babies in NCU	a. Mothers’ need for empathy from health care providers b. Mothers’ need of a safe space c. Mothers rely on family for logistical and financial support

### Theme 1: Mothers’ perceptions of quality of care were influenced by baby outcomes and health care providers’ responsiveness

#### a. Mothers’ confidence in medical treatment depended on baby outcomes

Overall mothers expressed a sense of assurance regarding the treatment the babies were receiving. This is because they observed that both their own and other people’s babies had improved following treatment. The success stories of mothers whose babies had recovered were frequently shared, giving mothers of newly admitted babies or those whose babies were still critically ill confidence.

*I recall how we arrived here; he was so small that he could not even cry or move. Within a few hours of being cared for, he began moving his hands and crying. And now you can see that he is gaining weight, that he is getting bigger, that the medicine is working.* (Mother of one, 20-year-old)

Even though most of them expressed a sense of confidence, mothers whose babies declined or did not recover quickly expressed a loss of faith in the treatment that their babies were receiving. Moreover, the babies who were deteriorating often required more medical treatment, and mothers frequently questioned the necessity of the numerous procedures and occasionally worried that these interventions might be harming their babies. It is important to note that, while there were many success stories in this NCU setting, most mothers had also witnessed a baby’s death in the NCU, which precipitated a lack of confidence in these mothers as well.

*Yes, I am concerned. Although the baby has little blood, the nurses are constantly pricking and drawing blood, and the condition remains unchanged. The baby started convulsing the other day after receiving a lot of drugs. The baby has been given so many drugs that it is now weak; they must have given it a lot of medication. I am sceptical of the care provided in this hospital.* (Mother of two, 25-year-old)

#### b. Health care providers’ responsiveness was not taken for granted

Whenever mothers believed that their baby was experiencing a life-threatening medical emergency, they would call the HCP for assistance. Some of the incidents commonly reported by the mothers were convulsions and babies gasping for air. If HCPs reacted swiftly to the mothers’ call for help, this was highly valued. Mothers were very appreciative of the HCPs who, despite being quite busy, would occasionally stop what they were doing to answer an urgent call.

*When compared to other wards I have visited, the NCU's health workers are quick to respond. Perhaps this is why my baby is still alive. When I told them (nurses) that my baby was having a problem, they rushed over to help. When I told them my baby was convulsing, they acted immediately. Even when I informed them that the oxygen was not flowing, they rushed to assist.* (Mother of three, 27-year-old)

However, mothers also interacted with unresponsive HCPs. When called upon, these HCPs would either not reply or would promise to come but never actually show up.

*These nurses here! The other time my child convulsed, I ran and told them to come and help; they said they were coming but then went about their business. I was terrified that my baby would die, but God came to my rescue.* (Mother of five, 32-year-old)

We also observed that some mothers believed that some babies received special treatment from HCPs in terms of responsiveness to their needs and time spent with them.

*In this unit, some babies get a lot of attention. Perhaps their mothers bribe the health workers? They (health care providers) are constantly checking on the baby, and whenever the mother calls, the health workers rush to see the baby. Meanwhile, they take up to an hour to arrive for me.* (Mother of six, 36-year-old)

### Theme 2: Mothers’ focus on “doing” things that aid their baby’s survival

Mothers felt a duty to make sure that their babies survived, and they therefore actively participated in their care. The activities mothers participated in included those that are routine like breastfeeding, cleaning the baby, and carrying the baby to the nurse for medication. Checking oxygen flow, removing the baby from oxygen when the power goes out because the supply is also cut off, and nasal gastric tube (NG tube) feeding were some of the additional tasks performed that needed a certain level of skill (as shown in Table 3).

**Table 3:** Activities mothers participated in while in the NCU

Routine interventions	More complex interventions
• Removing baby off oxygen when the power goes	• Breastfeeding
• Checking to see if oxygen is flowing in the prongs	• Wiping baby-bathing
• Notifying HWs when a baby has a danger sign	• Reminding nurses to give their babies medication
• Feeding in Nasal gastric tube	• Taking babies for medication
• Expressing milk	• Watching over the baby
• Using syringes to feed through the Nasal gastric tube	

Medical interventions: these are typically done by health care providers’ and require a level of medical judgment to be performed satisfactorily

The non-medical interventions are those that mothers typically do are non-invasive

*When you conceive and carry a pregnancy for nine months, it is your baby and your responsibility. I need to be involved in his care so that he can survive and live. That is my obligation as a mother and a woman; I cannot delegate it to anyone else, not even health workers. So, I do whatever needs to be done.* (Mother of five, 32-year-old)

The fact that there were very few HCPs in the NCU further catalysed the mothers’ need to take part in their baby’s care. The HCPs also encouraged mothers to help by taking time to train them to perform various tasks.

*The health workers are too few and the babies are just so many! They try to take time to empower us to look after our babies in a simple way. The nurses trained me how to express milk and the mills [amount] you are supposed to give the baby. I was also told that before expressing the milk, I need to wash my hands, then wash the cup with hot water, wash the syringe and they dry very well.* (Mother of four, 40-year-old mother)

Mothers stated that their main concern was helping the baby stay alive and leave the NCU. They appeared to be trying to avoid developing emotional attachments to their babies. Since the babies were so young, they hoped that they would have time to form a close bond with them later.

*Of course, I want my baby to recognize and bond with me as her mother. But that is not possible right now. It is not a priority; the baby needs to stay alive first. I am optimistic, because he is young, and when we get out of the hospital, we will bond, and she will recognize me as his mother. I had the opportunity to love, carry and breastfeed my previous babies, but not this one. She is on oxygen. She feeds through a tube* (Mother of three, 29-year-old mother).

### Theme 3: Mothers’ needs in the process of caring for babies in NCU

#### a. Mothers’ need for empathy from health care providers

Although mothers prioritized their babies and indeed wanted the HCPs to focus on them, they expressed a desire for empathy, since they were in what they termed as a “fragile” state. They were healing from giving birth and dealing with the unexpected experience of having a sick newborn baby. A lack of empathy was reflected in the negative response by HCPs when mothers, who were asked to carry out some tasks related to the care of their babies, were slow in doing so because of their condition.

*The health workers need to stop being angry at us. They should understand our situation. They should talk to us well because we are all human beings. Even if they instruct you to do something and you do not do it, they should understand. We are tired and sick as well* (Mother of two, 25-year-old).

However, there were various occasions when HCPs showed empathy to the mothers by paying attention to them, offering them words of encouragement, and being patient with them when they, for instance, did not bring their children for medication.

*I cried a lot. I had lost hope. The health workers comforted me and encouraged me to be strong. I was overwhelmed by thoughts. The health workers strengthened me. You cannot handle this period without someone to encourage you. Of course, for me, it was much better when the encouragement came from health workers as they know better* (Mother of six, 32-year-old).

There were several times when the mother reported that HCPs were rather cruel in the way they treated them. We observed that HCPs frequently shouted at mothers for not following NCU practices. Many mothers reported similar events. This happened to a great extent at night when HCPs were few and wanted to go to sleep.

*I was tired that day and went outside to rest on a mat; by the time I got up, it was after midnight, and the baby had missed his medication. I arrived just as the nurse had finished administering medication to the babies and had gone to sleep. I attempted to wake her up, but she refused. I was outside knocking, but she didn't seem to hear me. She later came out and inquired as to who was knocking. And I came over to explain myself. "Where were you?" she demanded angrily. I told her I had gone to bed because I was tired. She first argued, saying, "Will I always be there waiting for your babies?”* (Mother of two, 25-year-old)

#### b. Mothers’ need of a safe space

The NCUs are dormitory-like rooms with around 30 baby cots, three to four beds for Kangaroo Mother Care mothers, and a few chairs for mothers to sit on. Even though the nurses insisted that only mothers should stay in the NCU since they were breastfeeding the babies, additional family members were frequently present. Consequently, NCUs were often congested.

Mothers expressed worry that the environment was not safe. They were concerned that someone might steal their babies in this uncontrolled space. As a result, they were constantly nervous.

*I could not sleep because I had to keep an eye on my baby. I have not slept in days as you can see, I am exhausted. I could not go out to wash my clothes or bathe. There are so many people here, you cannot know their intentions. The mothers tell many stories of babies being stolen; it is very frightening.* (Mother of three, 28-year-old mother)

As mentioned in the theme of mothers’ perceptions of care, mothers felt it was their obligation to protect their babies, so they responded to their fear of having their baby stolen by always ensuring that a family member stayed to watch their baby whenever they left the NCU for any reason.

Besides the fear of babies being stolen, examples were given of theft of property in the NCU.

*There are no cameras. They steal everything. We are so many of us; you cannot differentiate who is a thief and who is not a thief. For example, they stole my money, and my phone. The man who stole it came and sat on a chair and I assumed he was someone’s husband. When I went out to the pharmacy, upon returning he was not there, and my phone was gone. Imagine some people send you money on the phone; I suffered with eating that whole week.* (Mother of five, 40-year-old).

#### c. Mothers rely on family for logistical and financial support

Mothers described the process of caring for the baby in the NCU as financially and physically draining. The common expenses included paying for medicines and laboratory tests that were not available in the hospital, buying food and tea, and sometimes extra baby clothes. Typically, it was the role of the spouse to take care of expenses. The provision of finances seemed to be what mothers primarily expected from them. We observed that some men were involved in other activities like watching over the baby in the NCU or washing clothes, but the women themselves rarely mentioned this, as the financial role seemed more relevant. It was common that the spouse would not have the money to cover all costs, so he got support from his family.

*My husband buys whatever is needed like diapers. Sometimes he fails to get all the money, his relatives support him. He is a teacher in a government school, and, understandably, he cannot raise all the money we need here. All his brothers and sisters have been very supportive.* (Mother of one, 21-year-old)

We noted from interviews that families that could afford these costs were happy to incur them and it was a source of satisfaction for the mothers, because then they felt that they did what was necessary to save their babies. However, it was challenging for those who could not afford it, who often felt bad about it, and their babies frequently missed treatment.

*I feel happy, we (her family) could afford to buy drugs and whatever is needed at NCU. I have made work easy for the health workers but also, I feel glad that I can provide everything needed for my baby to survive. I am lucky God has provided the money. I see other mothers struggling.* (Mother of four, 30-year-old)

Mothers also mentioned that caring for babies was physically exhausting. They did not rest much, and when they needed to rest, there were no beds in the NCU. They often had to go outside the NCU, or they slept on the floor at night. To cope with the demands, mothers depended on their families. Typically, the mother-in-law, sisters, sisters-in-law, and mothers entered the NCU to help with day-to-day care. They stayed at the hospital and supported mothers by watching over the baby, washing clothes, and going out to buy food or any other requirements.

For mothers who had no support from family, HCPs would mobilize resources to support them. The HCPs encouraged mothers to share resources.

*The other day I had spent all my money, I could not afford the medicine I needed to buy, and I had no food. The nurse asked one of the mothers who had an extra dose of medicine to give me, and she did. That nurse also asked all women to give me some food and tea that day. I was so thankful because my hands were tied that day. I was completely stressed out.* (27-year-old mother).

## Discussion

The study explored mothers’ perceptions and experiences of participating in the care of their sick newborn babies in the NCUs of two busy public hospitals in rural Uganda. The findings reveal that the mothers in the NCU prioritized their baby’s survival above all other concerns. Their confidence in HCPs and the effectiveness of the medication was largely based on their baby’s immediate outcome. Furthermore, the study highlighted the physical and finical challenges faced by mothers in the NCU. Inadequacies in the healthcare system, such as shortages of medicines and supplies, led to additional expenses for mothers and families. Due to understaffing, mothers were compelled to participate in care even in roles that were beyond their scope of competence. However, families, working together as a team, assisted mothers in dealing with the financial and logistical challenges of having a baby in the NCU.

This study demonstrates that the mothers’ primary concern was making sure their sick newborn babies stayed alive. Their concern about their babies’ survival may have been exacerbated by the NCU environment, where newborn deaths were frequent. Furthermore, we posit that mothers had limited confidence in the healthcare system, as their trust was contingent upon their baby’s outcome, which served as the primary indicator of quality care in their eyes.

The mothers in the study informed us that they put off creating emotional bonds with their babies because they were prioritizing “doing” things that ensured their baby’s survival. This postponement of bonding could potentially serve as a coping mechanism for mothers grappling with the fear of losing their babies. There are a number of studies that show that individuals deal with the fear of losing a baby by avoiding emotional attachment [26, 27]. For instance, some parents wait until their babies are older before naming them [28]. A community study in this setting indicated that the word “a thing,” which refers to a non-living item, is often used to refer to stillbirths and early newborn deaths [27].

Furthermore, it is also plausible that mental health problems like depression, stress, or anxiety, as supported by other studies, contributed to this difficulty in bonding [29, 30]. The study findings revealed that mothers experienced a great deal of stress as a result of their babies being admitted to the NCU. This stress stemmed from fears of their child’s death, concerns about the baby being stolen, and the financial costs associated with NCU stays. Thus, the mental state of these mothers likely played a role in hindering their ability to bond with their babies. This delayed bonding may have repercussions, as it could impede the achievement of certain benefits derived from a mother’s presence in the NCU.

The role of the NCUs is to care for sick babies and this was the focus of both staff and the mothers themselves. However, it is important to recognize that mothers were often sick after birth and were dealing with complex emotional needs. We contend that the practice of accommodating mothers in NCUs without designated resting areas or providing them with some basic care packages may have unintended consequences, as the well-being of mothers is closely linked to the well-being of their babies. These mothers are still at risk of complications such as postpartum bleeding and mental health issues like postpartum posttraumatic stress [31, 32]. Given that mothers spend a significant amount of time in the NCU attending to their babies, it becomes crucial to address aspects of their own care within the NCU environment to support their well-being. Embracing a mother-baby dyad approach within the NCU setting can bring about mutual benefits for both mother and baby [33]. For example, HCPs from the maternity ward could be asked to do a routine check on mothers in the NCU.

The study illustrates that the physical environment of the NCU is an important determinant of mothers’ negative experiences. The NCUs are congested, with many caregivers sharing the same small space. This arrangement increases the risk of property theft and also makes mothers feel concerned about their baby’s security. Mothers who give birth in high-volume public health facilities in Uganda have long expressed concern about their baby’s safety, and it has been identified as one of the reasons some mothers do not give birth in facilities [34]. Ensuring the safety of the babies in the NCU has however received little attention. While high-income settings have addressed this by establishing single or double-roomed NCUs, implementing such measures in Uganda may be impractical due to space and funding constraints in the short and medium term. Nonetheless, we believe that increasing the size of the NCU room, which is less expensive, and clearly designating a specific space for each mother-baby pair could be a good first step.

This study reveals significant policy and practice implications, the first being shedding light on the significant gaps found in essential quality-of-care standards established by the WHO [6]. Among the notable deficiencies identified, the study highlights the insufficient physical and emotional support provided to mothers in the NCU. These deficiencies can be traced back to underlying structural and systemic issues, including poor staffing, limited space, and inadequate availability of medicines. The presence of high caseloads, understaffing, and inadequate supplies creates a challenging environment where the delivery of family-centred, quality care becomes increasingly challenging. As emphasized by Koblinsky in an article, addressing the “obvious” structural problems like understaffing and lack of supplies is a crucial first step in improving the quality of care [35]. Thus, it is imperative that policy actors and health managers make it a top priority to address these critical factors, including staffing, supplies, and space, as the initial stride toward accomplishing family-centred newborn care.

Secondly, this study shows that mothers participated in tasks requiring a certain level of technical expertise, such as providing oxygen therapy and NG tube feeding. Despite their claims that the HCPs had trained them, we suspect that their brief instruction may not have been sufficient to prepare them for performing these tasks safely on their own. Therefore, it is crucial to specify which tasks mothers can perform. Striking the right balance between involving mothers in appropriate tasks and maintaining sufficient staffing levels is crucial for optimizing patient care and safety.

Third, the study demonstrates a “whole” family support approach where mothers are supported by many family members doing various tasks. The roles of family members in this context were gendered, with females including sisters, mothers, and mothers-in-law supporting with day-to-day hospital chores while males were tasked with finding financial assistance. This finding is similar to what Powis found in Senegal and refers to as a family “entourage” [36]. Understanding the gendered nature of these roles is crucial for programs that aim to involve families or men in newborn care. It is essential to take into account these gender norms and expectations in promoting newborn care and well-being.

This study’s strength lies in providing a rich understanding of mothers’ experiences and perceptions while caring for their babies in high-volume public health facilities in Uganda, capturing a variety of viewpoints through intentional heterogeneity in sampling. However, this study is not without limitations. We used three different interviewers; two had backgrounds in social science, and one had a background in medicine. These backgrounds may have influenced how the interviewers probed and/or phrased questions, which could have resulted in bias. However, to reduce this bias, data collectors were trained prior to interviews. In addition, we held daily debriefs to reflect on our interviews. The other limitation is that this study did not include private facilities, which are the biggest provider of care in Uganda. This is something that should be considered for future studies.

## Conclusion

The study shows that mothers’ top priorities in the NCU are the survival and well-being of their babies. This central focus shapes their evaluation of the quality of care their babies receive. However, systemic shortcomings such as medication shortages and understaffing compel mothers to take on additional, sometimes unfamiliar responsibilities, potentially jeopardizing newborn safety. Despite this, families show remarkable resilience by banding together to address the logistical and financial challenges associated with NCU care. To improve parental participation in resource limited NCUs, health system managers should prioritize measures that reduce financial burdens on families, address privacy and space concerns, as well as fully leverage families’ indispensable role in the care process. By implementing these strategies, health system managers can increase parental support and create an environment in NCUs that fosters optimal newborn care.

## Data Availability

The data sets used in the study are available from the corresponding author on reasonable request.
